# Understanding Metabolic Regulation Between Host and Pathogens: New Opportunities for the Development of Improved Therapeutic Strategies Against *Mycobacterium tuberculosis* Infection

**DOI:** 10.3389/fcimb.2021.635335

**Published:** 2021-03-16

**Authors:** Ji-Hae Park, Dahee Shim, Keu Eun San Kim, Wonsik Lee, Sung Jae Shin

**Affiliations:** ^1^Department of Microbiology, Institute for Immunology and Immunological Diseases, Brain Korea 21 Project for Graduate School of Medical Science, Yonsei University College of Medicine, Seoul, South Korea; ^2^School of Pharmacy, Sungkyunkwan University, Suwon, South Korea

**Keywords:** *Mycobacterium*, metabolism, immune cells, adjuvant therapy, host-directed therapy

## Abstract

*Mycobacterium tuberculosis* (Mtb) causes chronic granulomatous lung disease in humans. Recently, novel strategies such as host-directed therapeutics and adjunctive therapies that enhance the effect of existing antibiotics have emerged to better control Mtb infection. Recent advances in understanding the metabolic interplay between host immune cells and pathogens have provided new insights into how their interactions ultimately influence disease outcomes and antibiotic-treatment efficacy. In this review, we describe how metabolic cascades in immune environments and relevant metabolites produced from immune cells during Mtb infection play critical roles in the progression of diseases and induction of anti-Mtb protective immunity. In addition, we introduce how metabolic alterations in Mtb itself can lead to the development of persister cells that are resistant to host immunity and can eventually evade antibiotic attacks. Further understanding of the metabolic link between host cells and Mtb may contribute to not only the prevention of Mtb persister development but also the optimization of host anti-Mtb immunity together with enhanced efficacy of existing antibiotics. Overall, this review highlights novel approaches to improve and develop host-mediated therapeutic strategies against Mtb infection by restoring and switching pathogen-favoring metabolic conditions with host-favoring conditions.

## Introduction

Despite several decades of progress in controlling infectious diseases, tuberculosis (TB) remains the leading cause of death caused by pathogenic bacteria in humans (Floyd et al., 2018; WHO, 2019). TB is a chronic granulomatous disease, occurring mainly in the lungs (Elkard et al., 2016), and caused by the bacterium *Mycobacterium tuberculosis* (Mtb), which is pathogenic to humans. Mtb can be latent in the lung tissues of the host and persist throughout the lifetime of a patient. If the host immune response is disturbed, latent TB can be reactivated, which can lead to active transmission of the pathogen (Tufariello et al., 2003). During Mtb infection, host immune cells undergo significant metabolic alterations, and invoke a range of immune responses to defend against the infection (Shi et al., 2019a). The pathogen can survive in the host for a long time, using diverse pathways to escape the host immune system (Qualls and Murray, 2016). Investigating metabolic interactions between the host and mycobacterial pathogens is important in the development of strategies to strengthen the host defense system, and is critical for understanding the fundamental alterations which occur in the host immune system, from the establishment of infection to the disease outcome.

Recent advances in understanding host-pathogen interactions using multiple-omics analysis have provided insights into several pathways that may be novel therapeutic targets for TB treatment (Zimmermann et al., 2017; Serafini et al., 2019). Regulation of host anti-TB immunity, such as host-directed therapy (HDT) and adjunctive therapies, enhances the efficacy of existing antibiotics (Rayasam and Balganesh, 2015; Tobin, 2015). However, there are relatively few studies focusing on metabolic pathways that regulate host immune responses and boost antibiotic effects by effectively targeting Mtb. The development of new therapeutic strategies against Mtb infection requires an understanding of the immune cell functions and the major regulatory mechanisms of these immune functions (Liu et al., 2017).

We also need to understand the metabolic pathways that make Mtb refractory to anti-TB drugs. Bacteria can form persister cells by metabolic alteration, or induce drug resistance by genetic alteration of the bacteria in order to survive the stresses arising from the host environment and drug treatment (Jung et al., 2019). Persister cells, which are characterized by a decrease in the metabolism of the bacterium, are one of the causes of chronic infectious diseases, leading to the abuse of antibiotics (Jung et al., 2019) and the emergence of antibiotic-resistant bacteria (Defraine et al., 2018**)**. Therefore, understanding the mechanisms of Mtb persistence and resistance can lead to the development of effective strategies for antibiotic use, targeting bacterial metabolic pathways.

In this review, we address the immune metabolic mechanisms used by the host to control Mtb infection, and the mechanisms by which Mtb evades the host immunity. We focus on metabolic networks in which Mtb survives in human tissues by being refractory to anti-TB drugs. Based on this understanding, we propose a better control strategy for developing HDT against Mtb infection.

## Overview of Metabolic Interactions Between Host Immune Cells and Mtb

**Figure 1:**
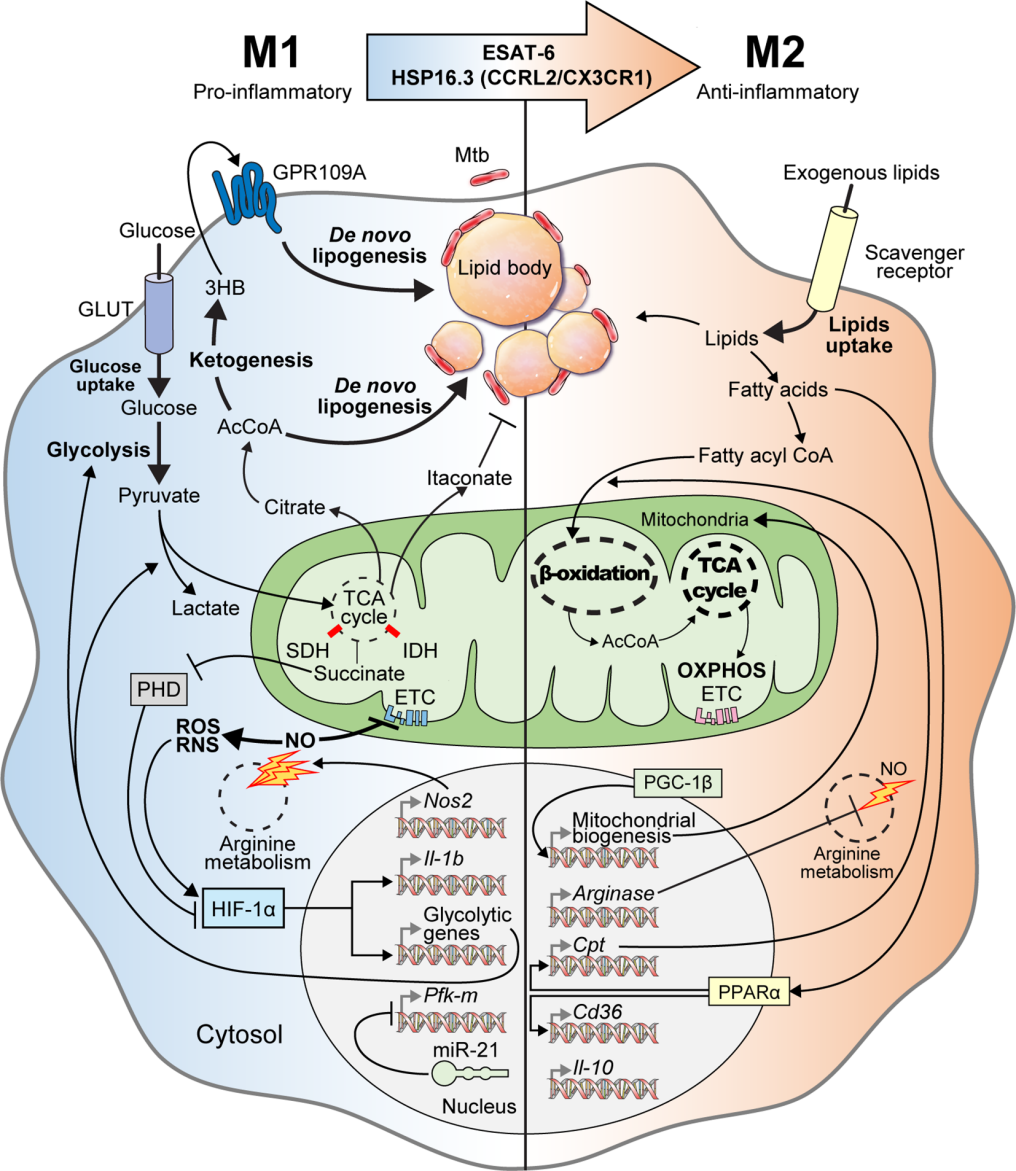
M1-to-M2 transition of Mtb-infected macrophages with metabolic reprogramming. In the early stage of Mtb infection, macrophages activate into pro-inflammatory M1 macrophages with metabolic reprogramming called the “Warburg effect.” Mtb-infected M1 macrophages have increased glycolytic capacity with excessive glucose entry through glucose transporters (GLUT). After glycolysis, glucose is degraded into pyruvate, which is used for the TCA cycle in mitochondria. Following M1 activation, the TCA cycle is blocked, which is accompanied by aconitate and succinate accumulation. Itaconate, which is driven by aconitate in Mtb-infected M1 macrophages, has anti-mycobacterial effects. Excessive succinate levels inhibit HIF prolyl-hydroxylases (PHD) and stabilization of HIF-1α. NOS2, a marker of M1 macrophages, is also expressed in Mtb-infected M1 macrophages to produce nitric oxide (NO) through arginine metabolism. Increased nitric oxide inhibits the ETC and activates reactive oxygen/nitrogen species (ROS/RNS). HIF-1α is also activated by ROS and RNS. Activated HIF-1α induces the transcription of glycolytic genes and *Il-1b* for boosting glycolysis, lactate production, and anti-mycobacterial immune responses, respectively. Intracellular Mtb induces miR-21 expression for inhibiting both glycolysis and IL-1β secretion by repressing *Pfk-m* transcription. When pyruvate is converted to citrate, citrate is further metabolized to acetyl-coenzyme A (AcCoA), which acts backbone for ketogenesis and *de novo* lipogenesis. During ketogenesis, D-3-hydrobutyrate (3HB) is generated from AcCoA and stimulates GPR109A for inducing *de novo* lipogenesis. Elevated *de novo* lipogenesis in Mtb-infected M1 macrophages generates the accumulation of lipid bodies, which are closely associated with intracellular Mtb. Thus, lipid-laden cells, which have a bubble-like morphology in the cytosol, are called “foamy” macrophages. In the late stage of Mtb infection, Mtb-infected M1 macrophages transition into anti-inflammatory M2 macrophages *via* mycobacterial components such as ESAT-6 and HSP16.3. These Mtb-infected M2 macrophages secrete anti-inflammatory cytokines such as IL-10 and TGF-β. Arginase, which is a marker of M2 macrophages, is also expressed in Mtb-infected M2 macrophages and acts on arginine metabolism to reduce NO generation. PGC-1β modulates mitochondrial biosynthesis to promote OXPHOS. PPARα induces the transcription of genes encoding lipid transporters and scavenger receptors to increase exogenous lipid utilization. Exogenous lipids are internalized by scavenger receptors in Mtb-infected M2 macrophages and generate free fatty acids, which are converted to fatty acyl CoA and translocated into the mitochondria in a carnitine palmitoyltransferase (CPT)-dependent manner. In Mtb-infected M2 macrophages, mitochondrial respiration, including β-oxidation and OXPHOS, are upregulated to maintain anti-inflammatory responses. Collectively, Mtb infection elicits metabolic reprogramming and modulates immune responses *via* M1-to-M2 macrophage transition.

Understanding immunometabolism and associated bioenergetic pathways is critical in elucidating the relationships between metabolic status and the functional roles of immune cells (Howard and Khader, 2020; Kornberg, 2020). Unlike other cells, immune cells must maintain homeostasis under normal physiological conditions, while being readily equipped to achieve a rapid and appropriate response during infection (Shi et al., 2016). Metabolic remodeling of the Warburg effect, characterized by increased glucose uptake and lactate production in the presence of oxygen in host immune cells, occurs in response to early Mtb infection (Cumming et al., 2020). The immune cells are characterized by the immune responses that occur during infection (Shi et al., 2019a). During early Mtb infection, classical M1 cells exhibit pro-inflammatory properties and T helper type 1 (Th1)-biased immunostimulatory properties that mediate antimicrobial defenses (Mantovani et al., 2004; Fan et al., 2015; Khan et al., 2019). M1 macrophages are characterized by the high production of pro-inflammatory cytokines [interleukin (IL)-1β, IL-6, IL-12, IL-23], antimicrobial peptides (cathelicidin, LL37), nitric oxide (NO), and reactive oxygen species (ROS) (Verreck et al., 2004; van der Does et al., 2010; Atri et al., 2018; Khan et al., 2019). In addition, M1 macrophages produce several inflammatory molecules that play an essential role in the host defense system, such as type I interferon (IFN), inducible nitric oxide synthase (iNOS), nitric oxide synthase 2 (NOS2), CXCL (1-3, 5-8), and CCL (2-5, 11) (Palomino and Marti, 2015; Bailey et al., 2019; Khan et al., 2019; Shi et al., 2019a). However, Mtb can survive and persist in the host by interfering with the host immune cell Warburg effect or by altering the M1/M2 polarization balance (Shi et al., 2016). In the early stages of Mtb infection, M1 polarization is dominantly involved, but as the infection progresses, the tricarboxylic acid cycle (TCA cycle) and oxidative phosphorylation (OXPHOS) in Mtb-infected macrophages are restored. Thus, M2 polarization eventually becomes a dominant characteristic (Shi et al., 2019a). The anti-inflammatory microRNA-21 (miR21) is induced, which limits glycolysis by inhibiting phosphofructokinase muscle (Hackett et al., 2020). IFN-γ has antagonistic effects against miR21, which supports host glycolysis to generate IL-1β. In addition, Mtb heat-shock protein 16.3 (Hsp16.3), a member of the α–crystal superfamily, is expressed during late Mtb infection. Hsp16.3 induced a M2-like phenotype in macrophages *via* CCRL2 and CX3CR1 and signal transduction of AKT/ERK/p38-MAPK (Zhang et al., 2020). The Early Secreted Antigenic Target 6 kDa (ESAT-6), which is a virulent Mtb factor, has been implicated in macrophage differentiation toward the M1 phenotype during early infection and the subsequent switch to an M2 phenotype during late infection (Refai et al., 2018). The M2 phenotype includes anti-inflammatory and angiogenic forms of non-classically activated macrophages that exhibit Th2-oriented regulatory immune properties (Khan et al., 2019). The polarization of M2 macrophages is mediated by anti-inflammatory cytokines such as IL-4, IL-10, and IL-13 (Martinez et al., 2009), and is characterized by the upregulation of several surface molecules such as dectin‐1, macrophage scavenger receptor (CD163 and CD204), CCR2, CXCR1, and CXCR2 (Gordon, 2003; Mantovani et al., 2004; Martinez et al., 2009; Khan et al., 2019) (**Figure 1**).

Mtb uses lipid bodies accumulated in macrophages and lactic acid produced by glycolysis as nutrient sources for their survival. Therefore, cholesterol synthesis, which produces a component of the lipid droplets (LD) required to maintain Mtb, may be a target to inhibit Mtb growth. In a recent study, simvastatin, a statin-based drug, showed anti-tubercular activity by inhibiting cholesterol biosynthesis (Bruiners et al., 2020). In another study, inhibiting the cholesterol biosynthetic pathway and mevalonate (MVA) pathway with statin drugs was proposed as an HDT for host protection against Mtb infection. (Parihar et al., 2014) (Chen et al., 2008). Therefore, studies targeting the host metabolism reprogrammed by Mtb infection may help in developing new HDT strategies.

Immune cells such as macrophages and T cells are important for anti-mycobacterial host defense, and tightly regulated T cell responses are fundamental to host survival against Mtb infection (Russell et al., 2019). The development of HDT for improving the regulation of immune metabolism in T cells has recently aroused considerable interest. In TB lesions, T cell mediated immunity is essential to the adaptive immune response, but the regulation of immune metabolism related to T cell response is less understood than the role of macrophages. The generation of adenosine triphosphate (ATP) in naïve T cells depends on oxidative phosphorylation (OXPHOS), and activated T cells reprogram their metabolism towards aerobic glycolysis to produce ATP (Buck et al., 2015). It has been reported that cyclophilin D (CypD), a mitochondrial matrix protein, affects T-cell metabolism and mitochondrial function. In CypD-deficient T cells, both glycolysis and OXPHOS were enhanced compared with control cells furthermore, there was increased generation of mitochondrial ROS. It is considered that increased ROS production leads to metabolic dysfunction, thereby increasing the proliferation of T cells. The inhibition of ROS generation with antioxidants reversed T cell proliferation to the control level. CypD inhibition increased the proportion of T cells with the phenotype of activated metabolism and enhanced proliferation, but the cells became highly susceptible to Mtb infection along with pulmonary immunopathology (Tzelepis et al., 2018). These results are contrary to the fact that the inhibition of CypD in macrophages reduced Mtb growth (Gan et al., 2005). Thus, intense T cell responses during Mtb infection are not necessarily beneficial, suggesting that dysregulated T cell responses may increase bacterial burden and susceptibility (Tzelepis et al., 2018). As Mtb infection progresses, CD8^+^ T cells impair mitochondrial function and increase dependence on glycolysis, but immune disruption occurs due to bioenergy deficiency. In a recent study, the expression of the inhibitory receptors PD-1 and CTLA-4 was significantly increased in Mtb-specific CD8^+^ T cells 12 weeks after H37Rv Mtb infection in mice. Analysis of the extracellular flux of CD8^+^ T cells revealed that CD8^+^ T cells were dysfunctional due to a metabolic “quiescence” state 12 weeks after infection (Russell et al., 2019). The use of metformin, developed as an anti-diabetic drug, in combination with anti-Mtb targeting drugs, restored the bioenergetic metabolism of CD8^+^ T cells in Mtb infected mice (Russell et al., 2019) and reduced the bacillary burden (Singhal et al., 2014). Therefore, it is possible to shorten the treatment period by improving the adaptive immune response of T cells to tuberculosis, through the development of HDT directed at T cell responses. In order to achieve this goal, greater knowledge of the adaptive immune response to TB is required (Cumming et al., 2020).

Additionally, Mtb may develop resistance to host immunity or antibiotic therapy *via* its own metabolism. Mtb metabolism provides diverse pathogenicity through various roles beyond replicative fuel, which helps maintain survival by resisting antibiotics and establishing chronic infection in host cells (Ehrt et al., 2018). Various lipid species present in the Mtb cell wall can be used to induce host pathological reactions, disrupt protective mechanisms or provide defense against antibiotic attacks. For example, by masking pathogen-related molecular patterns with phthiocerol dimycocerosates (PDIM) and suppressing Toll-like receptor 2 with sulfoglycolipids, innate immune signaling is inhibited. In addition, phosphatidylinositol mannosides (PIMs) contribute to the low permeability of the mycobacterial cell envelope and provide intrinsic antibiotic resistance (Dulberger et al., 2020). As such, the Mtb cell wall is regulated during human infection, thereby affecting the immune response and determining the sensitivity to antibiotics. Therefore, studying mycobacteria cell wall biosynthesis may help improve antibiotic sensitivity.

Another study revealed that mutation in tuberculosis transcription factor, *prpR*, in Mtb alters propionyl-CoA metabolism, which confers tolerance to the three most effective drugs: isoniazid, ofloxacin, and rifampin. Targeting gene alterations and subsequent metabolic mechanisms that affect Mtb drug sensitivity will help prevent tolerant strains and improve treatment outcomes (Hicks et al., 2018).

### Host Defense Mechanism by Glycolysis Metabolism of Macrophages Following Mtb Infection

When macrophages are activated to the M1 phenotype, they alter pro-inflammatory immune responses and metabolism such as the Warburg effect, which is characterized by a high glycolytic rate with increased lactate secretion in tumors and M1 macrophages (Warburg, 1956; Huang et al., 2014). In general, the major metabolic features in M1 macrophages are characterized by increased glycolytic flux and lactate formation, along with decreased pyruvate oxidation, TCA cycle activity, and oxidative phosphorylation, resulting in downregulated mitochondrial oxidative metabolism. This condition is responsible for *Hif1a* upregulation. HIF-1α is responsible for the Warburg effect, which promotes glycolytic flux by activating various genes encoding the main Warburg effect enzymes, such as glucose uptake transporter 1,6 (GLUT1,6), hexokinase 1,2 (HK1,2), the phosphofructokinase-1 (PFK-1) family, the phosphofructokinase 2 (PFK-2) family, 6-phosphofructo-2-kinase/fructose-2,6-biphosphatase 3 (PFKFB3), and the major lactate secretion transporter member 4 (MCT4, also known as SLC16A3) (Semenza et al., 1996; Nizet and Johnson, 2009; Semenza, 2010).

**Figure 2:**
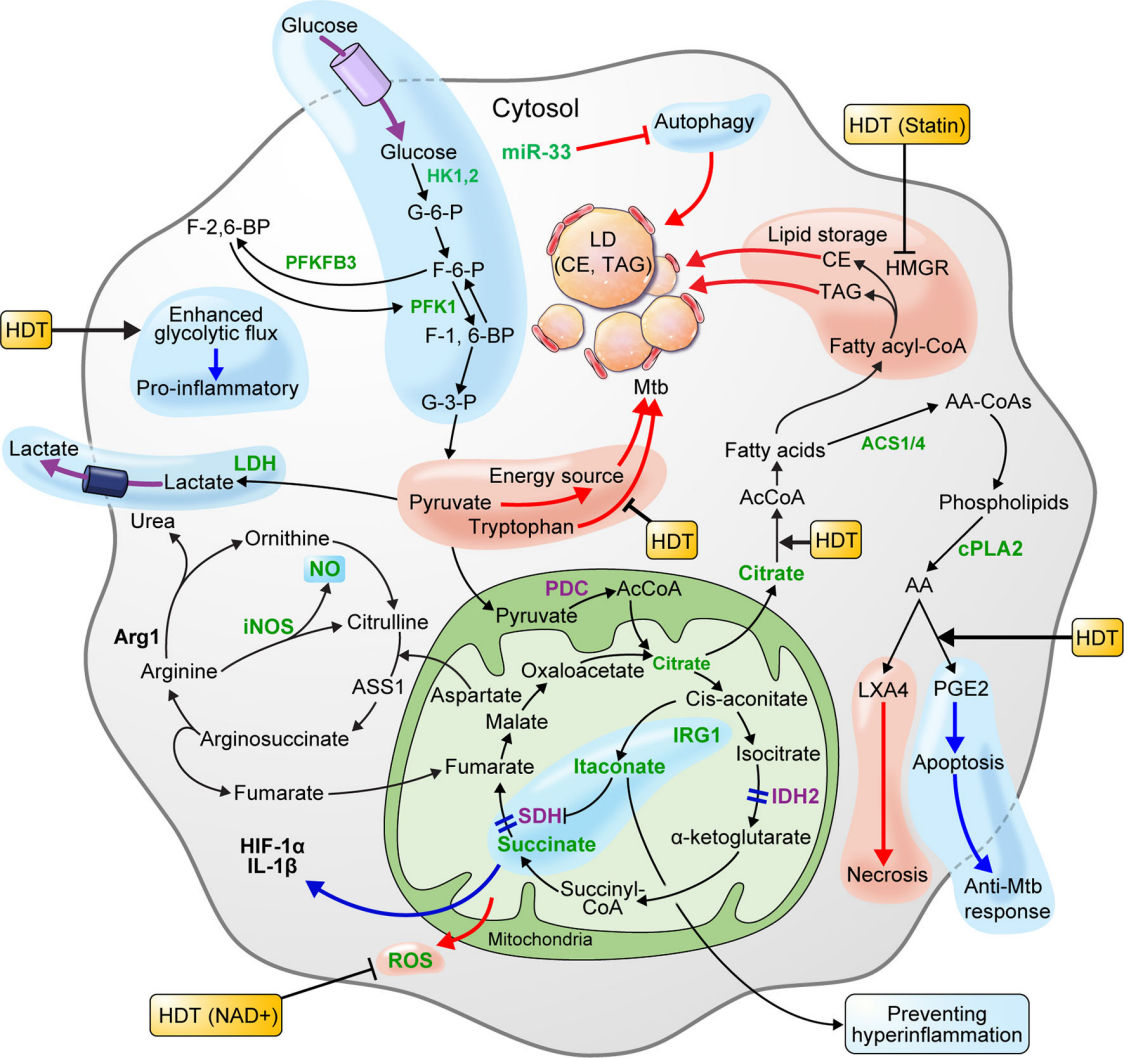
Metabolism of macrophages is beneficial to host defense or pathogen survival of during Mtb infection. HIF-1α, which is expressed in macrophage infected with Mtb, lowers the level of pyruvate by upregulating lactate dehydrogenase-A (LDH) and producing lactate. Pyruvate is a carbon source for Mtb and is used for proliferation. Thus, lowering the pyruvate level is advantageous for host defense. The glycolysis induced by Mtb infection limits Mtb survival through IL-1β induction. Elevated glycolytic flux and downregulated succinate dehydrogenase (SDH) in Mtb-infected macrophages triggers succinate accumulation. Succinate exhibits a pro-inflammatory reaction by inducing HIF-1α activation and IL-1β production. The pro-inflammatory mitochondrial ROS produced by succinate oxidation can limit necroptosis and Mtb replication by lowering ROS levels with nicotinamide adenine dinucleotide (NAD^+^) supplements. The enzymes participating in the TCA cycle and oxidative phosphorylation (OXPHOS) of macrophages infected with Mtb are downregulated. Itaconate inhibits SDH-mediated oxidation to increase succinate levels and induce IRG1-mediated anti-inflammatory responses. Arginase-1 (Arg1) contributes to the survival of pathogens in the early stages of infection, but controls infections during chronic infection. In macrophages, IDO-mediated tryptophan depletion induces immune tolerance, whereas inhibition of tryptophan synthesis using a specific gene deficiency in Mtb has a synergistic effect on Mtb growth inhibition. Macrophages infected with Mtb promote intracellular lipid metabolism to promote lipid droplet (LD) formation and differentiation into “foamy” macrophages, a characteristic of granulomas. This process is dependent on *de novo* cholesterol and fatty acid synthesis (FAS). Autophagy inhibited by the miR-33 locus blocks lipid catabolism and promotes cellular lipid accumulation. LD components, such as triacylglycerol (TAG) and cholesterol ester (CE), are nutrient sources for Mtb. Statins that inhibit cholesterol biosynthesis in hosts with chronic Mtb infection can be a host-directed drug target. Since the production of lipoxin A4 (LXA_4_) in Mtb-infected macrophages induces necrosis and prostaglandin E2 (PGE_2_) induces apoptosis, preferential PGE_2_ synthesis in the host may be an important host-directed therapy (HDT) for antimycobacterial responses. The blue pathway should be enhanced, and the red pathway weakened in favor of the host. In the immune metabolism of host cells infected by Mtb, increased expression and activity are green, and decreased expression and activity are purple.

In the early stage of infection, Mtb could elicit metabolic reprogramming of host cells, which is cognate with immunometabolism of M1 macrophages (Gleeson et al., 2016; Qualls and Murray, 2016). In metabolite studies of Mtb-infected macrophages, human alveolar macrophages, human monocyte-derived macrophages, and murine bone marrow-derived macrophages (BMDMs) showed MOI-dependent increased lactate production 3 h post-infection (Gleeson et al., 2016). Since lactate is produced from pyruvate reduction as the end product of glycolysis, increased lactate levels reflect an increase in intracellular glycolytic activity. Recent studies showed that HIF-1α expressed in Mtb-infected mouse BMDMs upregulates lactate dehydrogenase (LDH), an enzyme that converts pyruvate to lactate. In *Hif-1α* WT mouse BMDMs, pyruvate was converted to lactate by the increased LDH 12 h after Mtb infection, resulting in lower pyruvate concentrations. However, in *Hif1α* KO mice, pyruvate was not converted to lactate due to the absence of LDH, resulting in increased pyruvate levels. In addition, evidence that Mtb grows faster when pyruvate is the sole carbon source, rather than glucose, supports that pyruvate is utilized as an energy source for Mtb survival and replication. Therefore, lowering the pyruvate levels through increased LDH, which is upregulated by HIF-1α in macrophages, is crucial for the host defense mechanism against Mtb infection (Osada-Oka et al., 2019). Another recent study showed that HIF-1α activity is necessary for the control of pathological lung inflammation and long-term host survival during chronic Mtb infection (Braverman et al., 2016). Baay-Guzman et al. (2018) demonstrated the importance of HIF-1α activation in host defenses against Mtb by showing that blocking HIF-1α during early Mtb infection in BALB/c mice exacerbates the disease. Interestingly, it was shown that blocking HIF-1α with 2-methoxyestradiol during late TB infection eliminated the bacteria. By blocking HIF-1α during late TB, foam-like macrophages that resist apoptosis become susceptible to apoptosis, and the bacterial load is reduced (Baay-Guzman et al., 2018). However, even with a reduced Mtb load, HIF-1α plays a major role in regulating the pathological pneumonia during chronic Mtb infection. Thus, further study of HIF-1α blockade in late infection is necessary. In addition, treating murine BMDMs with 2-deoxyglucose (2DG), a glycolysis inhibitor, 24 h after Mtb H37Ra infection, significantly reduced the levels of IL-1β, a proinflammatory cytokine, and increased bacillary replication by macrophages (Gleeson et al., 2016). HIF-1α induces the transcription of IL-1β, a major pro-inflammatory cytokine (Tannahill et al., 2013), and mediates the Warburg effect, which is thought to contribute to the antimicrobial response during Mtb infection. Macrophages infected with the highly virulent pathogenic Mtb strain, H37Rv, showed increased glucose uptake. Further, the macrophage glycolytic flux induced by virulent Mtb is perturbed to synthesize ketone body D-3-hydrobutyrate (3HB) from AcCoA, which contributes to LD accumulation (Mehrotra et al., 2014). Similarly, macrophages at the center of lung granuloma of mice infected by Mtb showed a significantly lower Warburg effect than that of peripheral lung granuloma macrophages, but HIF-1α expression and glucose metabolism were higher. In summary, Mtb inhibits the immune activation of macrophages by reducing the Warburg effect and enhancing the macrophage glycolytic flux, thus funneling the carbon flux (Shi et al., 2016). Therefore, enhancing the Warburg effect, which is perturbed in macrophages during Mtb infection, may improve immune cell function. In addition, blocking HIF-1α using drugs such as 2-methoxyestradiol, which blocks HIF-1α in late TB infection, and co-treatment with antibiotics may improve the efficiency of TB treatment (**Figure 2**) (**Table 1**).

**Table 1:** Metabolic reprogramming in host cells during Mtb infection.

Pathway	Metabolite	Molecule	Model	Outcomes and interpretation	Effect on host immune cells	Proposed HDT strategy	Ref.
Glycolysis	Lactate		Human AM, human MDM, murine BMDM	Increased lactate Activation of intracellular glycolytic flux	Glycolysis upregulation is critical for host defense	LDH upregulated by HIF-α and induction of efficient Warburg effect are important in host immunity	(Gleeson et al., 2016)
	Lactate, pyruvate	HIF-1α, LDH	Mouse BMDM	Increased lactate, decreased pyruvate HIF-1α converts pyruvate to lactate *via* LDH enzyme	Pyruvate downregulation is critical for host defense		(Osada-Oka et al., 2019) (Shi et al., 2015)
			Supernatant fluid of Mtb-infected human primary cells and THP-1 cells	Decreased lactate, decreased pyruvate Decreased glycolysis metabolism in late infection	Destruction of host adaptive immune response by Mtb		(Cumming et al., 2018)
TCA cycle		SDH	HEK293 cells	Succinate accumulation by *sdh* inhibition	Induction of HIF-1α, Warburg effect and pro-inflammatory response	A treatment strategy is needed to prevent lung damage by controlling hyperinflammation through SDH inhibition	(Shi et al., 2019a), (Selak et al., 2005)
	Itaconate	IRG1	Mouse BMDM	Expression of itaconate and IRG1 Irg1 modulates inflammatory responses in the lung after Mtb infection	Irg1 is essential for host resistance to Mtb	Itaconate treatment can be a treatment strategy to prevent lung damage during chronic inflammation	(Nair et al., 2018)
		IRG1	Mouse BMDM	Increased itaconate, succinate Succinate increase by SDH-mediated oxidation inhibition by itaconate	IGR1-mediated anti-inflammatory response		(Lampropoulou et al., 2016)
		IRG1, ICL	Mtb culture	Itaconate inhibits Mtb ICL	Antimicrobial activity of macrophages		(Michelucci et al., 2013)
Mitochondria Respiration	ROS	TNT	Mtb infected THP-1 cells	ROS levels are up to 3 times increased Mitochondrial ROS is produced by dependence on TNT	Mitochondrial damage induced by TNT during macrophage necroptosis	ROS reduction by NAD^+^ supplementation is an HDT strategy to reduce necroptosis and limit Mtb replication	(Pajuelo et al., 2020)
Lipid	FFA and CL		PMA-differentiated THP-1 cells	Increased FFA and CL virMtb infection stimulates *de novo* synthesis of FFA and CL	Allow Mtb survival and persistence in the host	Inhibition of miR-33 locus expression, which contributes to LD accumulation in macrophages, and induction of PGE_2_ and LXB4 production support host immunity	(Mehrotra et al., 2014)
	Fatty acid β-oxidation	Micro RNA miR-33 locus in macrophage	THP-1 macrophage	Inducing the micro RNA miR-33 locus FAO damage by expression of micro RNA miR-33 locus by Mtb	Enhancement of lipid store in hosts preferred by mycobacteria		(Ouimet et al., 2016)
	Lipid droplet	IFN-γ, HIF-1α	Lung lesion of mice	IFN-γ signaling is required for LD formation during Mtb IFN-γ driven LD formation supports the production of host protective eicosanoids including PGE_2_ and LXB4	LDs support host immunity in Mtb infected macrophages		(Knight et al., 2018)
Amino acid	Arginine	Arg1	Mouse TB lung granulomas	Arg1 expression in hypoxic granulomas reduces T Cell proliferation	Arg1 inhibits bacterial growth in granulomas in TB	Arg1 expression is important for TB control in pulmonary granulomas	(Duque-Correa et al., 2014)
	Tryptophan, kynurenine	IDO	TB patient plasma	In MDR-TB, the IDO enzyme degrades tryptophan to increase kynurenine levels	Patients with high IDO levels are at higher risk for MDR-TB	Prevention of immune tolerance by blockade of tryptophan catabolism may be a strategy for HDT for TB	(Shi et al., 2019b)

AM, alveolar macrophages; Arg1, arginase1; CL, cholesterol; FAO, fatty acid oxidation; FFA, free fatty acids; HIF-1, hypoxia-inducible factor 1; ICL, isocitrate lyase; IDO, indole amine 2,3-dioxigenase; IFN, interferon; IRG1, immune-responsive gene 1; LD, lipid droplet; LDH, lactate dehydrogenase; LXB4, lipoxin B4; MDM, monocyte-derived macrophages; MDR, multidrug-resistant; Mtb, Mycobacterium tuberculosis; PGE_2_, prostaglandin E2; PMA, phorbol 12-myristate 13-acetate; SDH, succinate dehydrogenase; TB, tuberculosis; TCA cycle, tricarboxylic acid cycle; TNT, tuberculosis necrotizing toxin; virMtb, virulent Mycobacterium tuberculosis.

### TCA Cycle Metabolism-Mediated Host Defense Against Mtb Infection

The TCA cycle is a metabolic pathway in the mitochondrial matrix that catabolizes acetyl-coenzyme A (AcCoA) through cascade reactions of various enzymes. Citrate, the intermediate product of the TCA cycle, is converted to cis-aconitate by mitochondrial aconitase 2. Cis-aconitate is converted to itaconate by immune response gene 1 (IRG1) and to α-ketoglutarate by isocitrate dehydrogenase 2. In addition, succinate is oxidized to fumarate by succinate dehydrogenase (SDH) and converted to malate (Diskin and Pålsson-McDermott, 2018). When macrophages are stimulated by lipopolysaccharide (LPS) or other inflammatory signals, the accumulation of mitochondrial metabolites such as citrate, itaconate, and succinate occurs in the TCA cycle. This remodeling of the TCA cycle is a metabolic adaptation occurring in inflammatory macrophages that can lead to significant functional changes in immune cells (Lampropoulou et al., 2016; Diskin and Pålsson-McDermott, 2018).

During virulent Mtb infection, citrate, a TCA cycle intermediate, is released from the mitochondria into the cytosol (Mehrotra et al., 2014). Citrate is metabolized to AcCoA, which acts as a precursor of various inflammatory mediators of macrophages such as NO, ROS, and prostaglandin E2 (PGE_2_), which are related to macrophage activation (Infantino et al., 2011; Infantino et al., 2013; Infantino et al., 2014; Howard and Khader, 2020). In addition, citrate is used as a precursor of itaconate, which is an anti-inflammatory agent, and connects many metabolic and cellular processes, indicating that citrate metabolism is critical in the immune response (Williams and O’Neill, 2018).

Growing evidence suggests that Mtb-infected macrophages display disrupted TCA cycles leading to succinate accumulation, which functions as a signal linking metabolism and immunity (Tannahill et al., 2013; Mills and O’Neill, 2014; Shi et al., 2019a). Elevated glycolysis flux in early Mtb infection is due to succinate accumulation of (Tannahill et al., 2013; Mills and O’Neill, 2014). Succinate increases HIF-1α activity by inhibiting HIF prolyl hydrolase (Howard and Khader, 2020) and inducing IL-1β production (Tannahill et al., 2013), thereby limiting the anti-inflammatory response in activated macrophages. Succinate-induced IL-1β is crucial in controlling Mtb infection (Jayaraman et al., 2013; Gleeson et al., 2016; Ogryzko et al., 2019). In murine macrophages infected with Mtb, *sdh* was downregulated, which may contribute to succinate accumulation (Shi et al., 2019a; Howard and Khader, 2020). Itaconate, which is overexpressed in Mtb-infected macrophages, acts as an anti-inflammatory factor that inhibits SDH-mediated succinate oxidation (Shin et al., 2011; Diskin and Pålsson-McDermott, 2018). Recent studies have identified SDH modulators that play an important role in determining the inflammatory phenotype. Treatment with dimethyl malonate (DMM), a competitive inhibitor of SDH-mediated succinate oxidation, produces malonate inside macrophages and results in increased succinate levels without changing HIF-1α and IL-1β levels (Nonnenmacher and Hiller, 2018). In addition, BMDMs from C57BL/6 mice treated with LPS demonstrated attenuated IL-1β activity and increased IL-10 production. Therefore, SDH inhibition by DMM results in an anti-inflammatory effect (Mills and O’Neill, 2014; Mills et al., 2016; Mills and O’Neill, 2016). In addition, succinate oxidation by SDH can lead to pro-inflammatory mitochondrial ROS production. Inhibiting ROS production with rotenone can inhibit the inflammatory phenotype (Mills et al., 2016). Thus, inhibiting succinate oxidation with itaconate or DMM could be a potential treatment to control Mtb infection and immune-mediated tissue damage through inhibition of ROS production (**Figure 2**) (**Table 1**).

Itaconate, which contributes to succinate accumulation, is produced by immune response gene1, which is activated by Mtb infection (Nair et al., 2018). Indeed, itaconate is an antibacterial metabolite that inhibits isocitrate lyase activity, which supports the growth of bacterial infections (McFadden and Purohit, 1977). In addition to its antibacterial function, itaconate inhibits mitochondrial respiration and inhibits pro-inflammatory cytokines such as IL1-β, IL6, and IL12p70 in macrophages *in vivo* and *in vitro* (Lampropoulou et al., 2016). In another study, *Irg1-*knockout mice display high inflammatory cytokine production and severe lung disease during Mtb infection, thus highlighting the importance of *Irg1* in host immunity. In other words, the ability to modulate inflammation by *Irg1* expression in immune cell metabolism can suppress excessive immune responses, thereby reducing lung disease during infected with Mtb (Nair et al., 2018). Taken together, succinate-mediated proinflammatory responses are induced by itaconate overproduction, while itaconate-mediated anti-inflammatory responses are produced by IRG1 during Mtb infection, thus preventing damage to host cells from hyperinflammation (Shi et al., 2019a). Therefore, to limit TB progression from a pro-inflammatory state to a chronic inflammatory state, a treatment strategy that prevents lung damage by eradicating Mtb and revealing the degree of inflammation of TB patients is needed (**Figure 2**) (**Table 1**).

### Mitochondrial Respiration of Macrophages Following Mtb Infection

In the mitochondrial TCA cycle, pyruvate and fatty acid are oxidized, and nicotinamide adenine dinucleotide (NAD^+^) is reduced to nicotinamide adenine dinucleotide hydride (NADH). Then, NADH is used during OXPHOS to produce ATP (Cortassa et al., 2019). During Mtb infection and M1 macrophage polarization, Mtb secretes NAD^+^ glycohydrolase to deplete NAD^+^, causing necroptosis of infected cells (Pajuelo et al., 2018). Additionally, Mtb-infected macrophages express nicotinamide phosphoribosyltransferase to maintain homeostasis by increasing NAD synthesis. Nicotinamide phosphoribosyltransferase is regulated by SIRT6, and SIRT6 prevents excessive inflammatory responses during Mtb infection and prolongs macrophage survival (Shi et al., 2019a; Sociali et al., 2019; Howard and Khader, 2020). In the early stages of Mtb infection, respiratory depression and OXPHOS-related enzymes are downregulated, and decreased OXPHOS results in ROS and reactive nitrogen species (RNS) production (Shi et al., 2015; Howard and Khader, 2020). The origin of ROS and RNS oxidative stress can be derived from several metabolic processes. In Mtb-infected murine macrophages, electron transport chain (ETC) and mitochondrial function are inhibited by NO generated by highly-expressed NOS2, which may lead to ROS overproduction. Additionally, NO, which is produced by iNOS/NOS2, can react with $O_{2}^{-}$ to form stronger RNS like ONOO^-^ (Shi et al., 2019a). RNS also inhibit ETC and mitochondrial function, reducing redox and increasing ROS (Beltrán et al., 2000; Everts et al., 2012).

Increased oxidative stress may be a pro-inflammatory response to combat Mtb infection, but conversely, Mtb may possess diverse mechanisms that promotes bacterial spread and replication by inducing macrophage necrosis (Lerner et al., 2017). The TCA cycle and OXPHOS are upregulated in murine macrophages 24 h post-Mtb infection. Ultimately, oxidative metabolism may be increased at a later time-point during Mtb infection (Howard and Khader, 2020). OXPHOS upregulation is driven by *Pgc1b* induction, which encodes peroxisome proliferator-activated receptor gamma-coactivator-1β (PGC-1β), a key transcriptional factor in mitochondrial biogenesis and promotes oxidative metabolism. Transgenic PGC-1β expression primes M2 macrophage activation and inhibits pro-inflammatory cytokine production (Lin et al., 2005; Vats et al., 2006). Inhibiting M1 polarization represents the transition of Mtb-infected BMDMs to the adaptation/resolution stage, which may be a mechanism for Mtb to survive by avoiding the host immune system (Shi et al., 2019a). In a recent study, it was shown that catalytic activity of TB necrotizing toxin induces mitochondrial ROS in Mtb-infected macrophages, causing necroptosis and promoting Mtb replication. Reducing ROS levels with NAD^+^ supplementation protected macrophages from cell death and restricted mycobacterial replication. Taken together, the immunological function of ROS production in macrophages during Mtb infection plays multiple roles, but combining the antioxidant N-acetyl-cysteine with nicotinamide for NAD^+^ supplementation could enhance antibacterial TB chemotherapy (Pajuelo et al., 2020) (**Figure 2**) (**Table 1**).

### Formation of LD-Rich Foam-Like Macrophages Supports Mtb Persistence Due to Perturbed Lipid Metabolism in Mtb-Infected Macrophages

Macrophages infected with Mtb increase lipid metabolism, which results in lipid droplet (LD) formation and further differentiation into “foamy” macrophages within granulomas (Hunter et al., 2007; Peyron et al., 2008; Shim et al., 2020). Mtb and other bacteria are believed to specifically induce LD formation as a pathogenic strategy for use as a carbon source to promote intracellular growth (Saka and Valdivia, 2012; Nolan et al., 2017). Mtb escapes to the cytosol during macrophage infection and uses cholesterol and fatty acids (FAs) contained in lipid droplets as a nutrient source. The Mtb triacylglycerol (TAG) composition is almost the same as that of the host because Mtb causes TAG accumulation in host cells (Daniel et al., 2011).

The accumulation of LDs in Mtb-infected macrophages depends on the induction of *de novo* cholesterol and fatty acid synthesis (FAS) by host cells (Mehrotra et al., 2014). Reduced isocitrate dehydrogenase 2 levels in mitochondria cause metabolic breakpoints between isocitrate and α-ketoglutarate production in the TCA cycle, resulting in citrate accumulation (Jha et al., 2015). Citrate is transported from the mitochondria to the cytosol by SLC25A1, where it is metabolized to AcCoA and converted to MVA and malonyl-CoA to support cholesterol and free fatty acid synthesis, respectively (Mehrotra et al., 2014). In addition to *de novo* lipogenesis, LD accumulation also contributes to the inhibition of lipolysis in Mtb-infected macrophages. 3-hydroxybutyrate (3HB), which is an end product of ketone body synthesis that is supplemented with excessive glycolysis during Mtb infection, is a G-protein coupled receptor GPR109a agonist. ESAT-6 protein secreted by H37Rv-infected macrophages stimulates glucose uptake. This stimulation increases the glycolysis rate and induces AcCoA accumulation, and then produces 3HB through the 3-hydroxy-3-methylglutaryl coenzyme A cycle reaction. Secreted 3HB inhibits adenylate cyclase through GPR109A activation, which reduces cAMP levels, weakens PKA activity, and reduces perilipin phosphorylation. Non-phosphorylated perilipin forms a protective coating on the LD surface, protecting it from lipolysis by hormone-sensitive lipase, eventually causing LD accumulation in macrophages (Singh et al., 2012). In other words, the activation of GPR109A by 3HB imparts anti-lipolytic abilities leading to perturbed lipid metabolism and LD accumulation in Mtb-infected macrophages in an ESAT-6 dependent manner. Inhibiting GPR109A with mepenzolate bromide effectively reduces the intracellular Mtb bacillary load, LD accumulation in alveolar macrophages, and the number of granulomas *in vivo* (Verma et al., 2019). Indeed, mepenzolate bromide inhibits the 3HB/GRP109A feedback loop activated by intracellular Mtb, thereby interfering with LD accumulation in macrophages. Thus, mepenzolate bromide is a candidate anti-TB drug targeting the host lipid metabolism pathway (**Figure 1**).

In addition to perturbations in lipid metabolism induced by Mtb-induced GPR109A activation, a mechanism of autophagy evasion through miR-33 expression has been reported. Inducing miR-33 expression inhibits autophagy and reprograms host lipid metabolism for increasing intracellular bacterial loads. Autophagy plays a role in promoting lipid catabolism by delivering TAG and cholesterol esters stored in LDs to lysosomes, whereas autophagy inhibited by the miR-33 locus blocks lipid catabolism and promotes cellular lipid accumulation. Eventually, Mtb induces miR-33 expression during macrophage infection to inhibit autophagy, thereby avoiding bacterial degradation (xenophagy) and accumulating LDs that provide a nutrient-rich environment for replication. In contrast, genetic and pharmacological miR-33 silencing promotes AMPK-dependent activation of FOXO2 and transcription factor EB, which engages lipid catabolism. Thus, suppressing miR-33 expression locus in host cells could be a strategy to inhibit Mtb survival by maintaining autophagy, lysosome function, and lipid homeostasis, which are also crucial for host innate immunity (Ouimet et al., 2016). Taken together, understanding the immunometabolism of foamy macrophages is necessary to establish the novel HDT strategies that inhibit LD accumulation and boost anti-TB immune responses to improve TB treatments (**Figure 2**) (**Table 1**).

### Regulation of Immune Responses by Arginine Metabolism of Mtb-Infected Macrophages

iNOS expressed in pro-inflammatory macrophages catalyzes NO production using arginine as a substrate (Mattila et al., 2013). NO is a major anti-mycobacterial molecule, and in addition to microbicidal activity, it regulates IFN-γ-mediated anti-Mtb activity and inflammatory responses during infection (Lee and Kornfeld, 2010; Herbst et al., 2011). The host protective immunity against TB releases IFN-γ from T cells to induce NOS2 expression and enhance NO production, which prevents Mtb growth and subsequent inflammatory responses (Jamaati et al., 2017). In addition, inhibition of the nod-like receptor family, pyrin domain containing 3 (NRP3) inflammasome factor by NO plays a role in preventing Mtb growth and subsequent pathology by reducing IL-1β expression, eventually preventing neutrophil recruitment to infection sites (Mishra et al., 2017). TB patients are deficient in L-arginine, the NO precursor, and vitamin D, which emphasizes the importance of NO in the development of TB (Ralph et al., 2008). Because NO bioavailability is significantly lower in people with severe TB, increasing NO delivery to the lungs of pulmonary TB patients may decrease infectivity in people with drug-resistant TB (Ralph et al., 2013; Jamaati et al., 2017). Therefore, developing a method to deliver high levels of NO to macrophages may prevent excessive inflammation in lungs infected with Mtb in TB patients (**Figure 2**).

Arginase 1 (Arg 1) is expressed in anti-inflammatory macrophages, where it competes with NOS for arginine to produce ornithine, which is synthesized as proline, and can promote collagen synthesis (leading to fibrosis) in tuberculous granulomas. Arg1 is primarily present in M2 macrophages localized around granulomas (Mattila et al., 2013) and is associated with the host anti-inflammatory response (Yang and Ming, 2014). In addition, inducting Arg1 expression in macrophages may inhibit antimicrobial activity by downregulating NO and reactive RNS production (Kumar et al., 2019). Conversely, other studies have suggested that Arg1 may play a crucial role in host protection by regulating inflammation and necrosis in hypoxic granulomas (Duque-Correa et al., 2014). Because TB granulomas are often hypoxic, killing bacteria through NOS may not be optimal. However, arginine metabolism using Arg1 does not require oxygen and may play an important role in controlling TB in pulmonary granulomas that occur during Mtb infection. In a TB murine granuloma model without NOS2, Arg1 contributed to the inhibition of T cell proliferation in the hypoxic granuloma region, reduced the incidence and expansion of necrosis, and controlled Mtb growth and pathology (Duque-Correa et al., 2014). Thus, in hypoxic conditions such as TB granulomas, Arg1 likely plays a key role in Mtb control. In other words, for a protective immune response to Mtb, pro-inflammatory macrophages with bactericidal activity and anti-inflammatory macrophages that limit immunopathology are required. Therefore, to apply NOS or Arg1, which regulate the inflammatory macrophage response, as HDT targets, the inflammatory state and lung pathology of TB patients must be considered (**Figure 2**) (**Table 1**).

### Immune Suppression by Tryptophan Catabolism in Host Cells Infected With Mtb

As another pathway that regulates host inflammation and immunity, tryptophan metabolism has also been extensively studied. Tryptophan metabolism regulates hyperinflammation and induces long-term immune tolerance (Sorgdrager et al., 2019). The enzyme indoleamine 2,3-dioxygenase (IDO), which catalyzes the first and rate-limiting step of conversion from tryptophan to kynurenine, has been studied in terms of various inflammatory diseases (Moffett and Namboodiri, 2003; Sorgdrager et al., 2019; Gostner et al., 2020). IDO is activated in response to IFN-γ and Th1 cytokines released during inflammation, creating a local or systemic environment with high kynurenine and low tryptophan, and altering the function of neighboring cells (Sorgdrager et al., 2019). Tryptophan catabolism plays a pivotal role in regulating the immune response through a mechanism that slows T cell proliferation by reducing the tryptophan supply (Moffett and Namboodiri, 2003). Moreover, kynurenine triggers regulatory T cells (Treg) development, while 3-hydroxyanthranilic acid and quinolinic acid inhibit specific immune cells by selectively inducing Th1 cell apoptosis (Fallarino et al., 2002; Moffett and Namboodiri, 2003). In summary, tryptophan catabolism can promote immune tolerance through mechanisms that inhibit T cell proliferation and promote apoptosis, and IDO activity can contribute to immune suppression in patients with an activated immune system, especially in chronic disease states (Moffett and Namboodiri, 2003). In fact, plasma kynurenine levels are high and tryptophan levels are low in patients with MDR-TB, and the risk of MDR-TB was higher as the plasma IDO level increased (Shi et al., 2019b). In addition, in macaques with suppressed IDO activity, host survival was increased by reducing bacterial burden, pathology, and clinical signs of TB. This increased protection was accompanied by the translocation of more T cells to the lesion core within the granuloma organization. In summary, the inhibition of IDO activity enables T cells to access the lesion core and alters the granuloma organization, thereby promoting bacterial killing (Gautam et al., 2018). In other words, preventing immune tolerance by blocking tryptophan catabolism in the host may be a new HDT strategy for clinical application of immunotherapy in TB treatment. In addition, IDO-mediated tryptophan depletion may have anti-microbial properties against pathogens that may require tryptophan nutrients (Munn and Mellor, 2013). However, since Mtb synthesizes tryptophan on its own, IDO-mediated tryptophan depletion does not have a direct effect on anti-bacterial activity (Zhang et al., 2013).

When intracellular IDO is induced by CD4^+^ T cell-mediated IFN-γ in response to Mtb infection, intracellular tryptophan is reduced. In this case, Mtb synthesizes mycobacterial tryptophan to maintain survival in macrophages. The *trpE*-deficient Mtb is particularly sensitive to CD4^+^ T cell-mediated stress, thereby inhibiting bacterial growth. The combined effect of 2-amino-6-fluorobenzoic acid (6-FABA), a small molecule that inhibits TrpE, and IFN-γ showed clear 40-fold synergy in mice and nine-fold synergy in human macrophages (Zhang et al., 2013). As such, tryptophan depletion, which is a host immune response mechanism, and disruption of tryptophan synthesis using a specific gene deficiency in Mtb can synergistically inhibiting Mtb growth. Thus, this combined approach may be an effective method for TB treatment (**Figure 2**) (**Table 1**).

## Metabolic Pathways in Mtb and Implications for Survival Tactics

Understanding of the metabolic alterations in Mtb has progressed recently, and much information has been reported on the maintenance of Mtb cell wall biosynthesis (Daffe et al., 2014; Jackson, 2014; Jankute et al., 2015), energy metabolism and respiration (Cook et al., 2014; Cook et al., 2017), central carbon metabolism (Baughn and Rhee, 2014; Ehrt et al., 2018), nitrogen metabolism (Gouzy et al., 2013a; Gouzy et al., 2013b; Gouzy et al., 2014a; Gouzy et al., 2014b), sulfur metabolism (Hatzios and Bertozzi, 2011; Zeng et al., 2013), metallobiology (Marcela Rodriguez and Neyrolles, 2014; Chao et al., 2019) and nucleic acid metabolism (Ditse et al., 2017).

According to a recent study, one metabolic pathway that is essential for Mtb growth is aspartate synthesis coupled to glutamine-mediated assimilation. Rv3722c serves to balance anaplerosis and cataplerosis of the Mtb TCA cycle and is involved in the transfer of assimilated nitrogen from glutamate to aspartate. Metabolites that are dependent on aspartate are inosine-5’-monophosphate and AMP, which are related to purine metabolism. *In vitro*, aspartate supplementation in Mtb lacking Rv3722c restores bacterial metabolic activity and growth, and Mtb growth is further increased when hypoxanthine, a purine salvage pathway intermediate, is added. Rv3722c is the primary Mtb aspartate amino transferase (AspAT) and is important for Mtb growth (Jansen et al., 2020).

In addition, bacterial pathogens, including Mtb, use various adaptive strategies to survive during antibiotic treatment (Eisenreich et al., 2015; Ehrt et al., 2018). For example, trehalose is a core component of the Mtb cell surface, but MDR-Mtb has a mechanism to maintain ATP levels by biosynthesizing central carbon metabolism intermediates using trehalose as an internal carbon source. Thus, by remodeling trehalose metabolism, Mtb can enter a drug-resistant state and reduce the efficacy of bedaquiline (BDQ), a species-selective Mtb ATP synthase inhibitor (Lee et al., 2019). Trehalose can be regenerated through a recycling pathway that turns over the cell wall glycolipid trehalose monomycolate. This process is necessary to establish infection in mice by reprograming Mtb lipid metabolism. As such, Mtb uses intracellular and extracellular carbon sources for growth, replication, and antibiotic resistance. Various metabolic regulation mechanisms have been proposed during infection (Ehrt et al., 2018) (**Table 2**).

**Table 2:** Metabolic strategies of Mtb for survival within macrophages.

Category	Bacterial factors	Biological process	Molecular function	Implications	Ref.
Survival factors	Central carbon metabolism	Glycolysis, PPP, TCA cycle, glyoxylate shunt, methylcitrate cycle, gluconeogenesis	Core feature to provide energy	The main metabolic network that sustains Mtb survival	(Baughn and Rhee, 2014) (Hards et al., 2020)
Virulence factors	Various genes and proteins	Lipid and fatty acid metabolism, Cell envelope proteins, proteins inhibiting antimicrobial effectors of the macrophage, protein kinases, proteases, metal-transporter proteins, gene expression regulators	Evolution of various virulence factors to modulate host immune response	Essential bacterial genes/proteins for the virulence of MTBC species	(Forrellad et al., 2013) (Zondervan et al., 2018) (Orgeur and Brosch, 2018)
Growth factor	Rv3722c	Aspartate-dependent nitrogen metabolism	Rv3722c as primary aspartate aminotransferase mediates nitrogen distribution	The gene *rv3722c* is essential for Mtb growth	(Jansen et al., 2020)
Drug resistance factor	TreS	Trehalose metabolism remodeling	PLB and MDR-Mtb use trehalose to maintain ATP levels	Reduce the efficacy of BDQ by remodeling trehalose metabolism	(Lee et al., 2019)
	*glpK*	Glycerol-3-kinase required for glycerol catabolism	Variation in the *glpK* coding sequence produces a drug-tolerant phenotype	Reduction of antibiotic efficacy and resistance by metabolic mutation of glycerol catabolism	(Bellerose et al., 2019)
	*prpR*	Alteration of propionyl-CoA metabolism	*prpR* enriched in drug resistant strains	Confers conditional drug tolerance of *prpR* mutations by altering propionyl-CoA metabolism	(Hicks et al., 2018)
	PptT (encoded by *rv2794c*) PptH (encoded by *rv2795c*)	PptT is involved in AcCoA metabolism to synthesize cell wall lipid	PptH mutations that deactivate the PptT reaction cause antimycobacterial resistance	Inducing antibiotic resistance mechanism through self-destructive reaction of Mtb	(Ballinger et al., 2019)
	*tgs1*	Accumulation of TAG	Quiescent metabolic activity by limitation of TCA cycle activity	Mtb of drug-resistant persister cells with accumulated TAG	(Garton et al., 2008; Shi et al., 2010; Baek et al., 2011)
Immune regulation factor	TDM, Ac2SGL, PIM, LM	Rich cell wall lipids	Enhanced immunopathology	Modulation of host immune pathological response by Mtb cell wall lipid	(Queiroz and Riley, 2017)
	PDIM, MA, SL-1, LAM, Man-LAM, DAT, PAT		Dampened immunopathology		
LD accumulation in macrophage for Mtb persistence	Mtb induces FM formation in macrophage	Mtb converts the glycolytic pathway of host cell metabolism into 3HB synthesis Accumulation of LD by anti-lipolytic ability of 3HB	LDs serve as nutrients and secure niche for Mtb	Targeting host lipid metabolic pathways perturbed by Mtb may provide TB chemotherapy	(Singh et al., 2012)
	Mtb modulates autophagy and LD accumulation	miR-33, expressed during macrophage infection by Mtb, inhibits autophagy, lysosomal function, and FAO	Mtb persists by avoiding lysosome degradation and establishing a lipid riche niche		(Ouimet et al., 2016)
Mtb factor using host metabolites as nutrients	Rv2498c	Dissimilation of itaconate to produce AcCoA and pyruvate Catabolism of leucine to produce AcCoA and acetoacetate	Rv2498c as a bifunctional β-hydroxyacyl-CoA lyase	MTB mechanism for resistance to itaconate, an antimicrobial agent in the host and a modulator of the inflammatory response Using host-derived molecules as nutrients by functional enzyme in Mtb	(Wang et al., 2019a)

3HB, 3-hydroxybutyrate; Ac_2_SGL, diacylated sulphoglycolipid; ATP, adenosine triphosphate; BDQ, bedaquiline; CoA, coenzyme A; DAT, diacyltrehalose; FAO, fatty acid oxidation; FM, foamy macrophage; LAM, lipoarabinomannan; LD, lipid droplet; LM, lipomannan; MA, mycolic acids; Man-LAM, mannose-capped lipoarabinomannan; MDR, multidrug-resistant; MTBC, Mycobacterium tuberculosis complex; PAT, polyacyltrehalose; PDIM, phthiocerol dimycocerosate; PIM, phosphatidyl inositol; PLB, Mtb persister-like bacilli; PPP, pentose phosphate pathway; PptH, ppt hydrolase; PptT, phosphopantetheinyl transferase; SL-1, sulpholipid-1; TDM, trehalose dimycolate.

### Mycobacterial Metabolism for Survival Strategies in Host Target Cells

Mycobacterial central carbon metabolism is a major determinant of virulence (Weiner et al., 2018). The lipids that comprise the bacterial cell wall are sufficient to activate the host immune response (Queiroz and Riley, 2017). In addition, for *Mycobacterium* to cause disease in the host, its metabolism must be reprogrammed to resist host defense mechanisms and to gain nutrients from the host (Beste et al., 2013).

**Figure 3:**
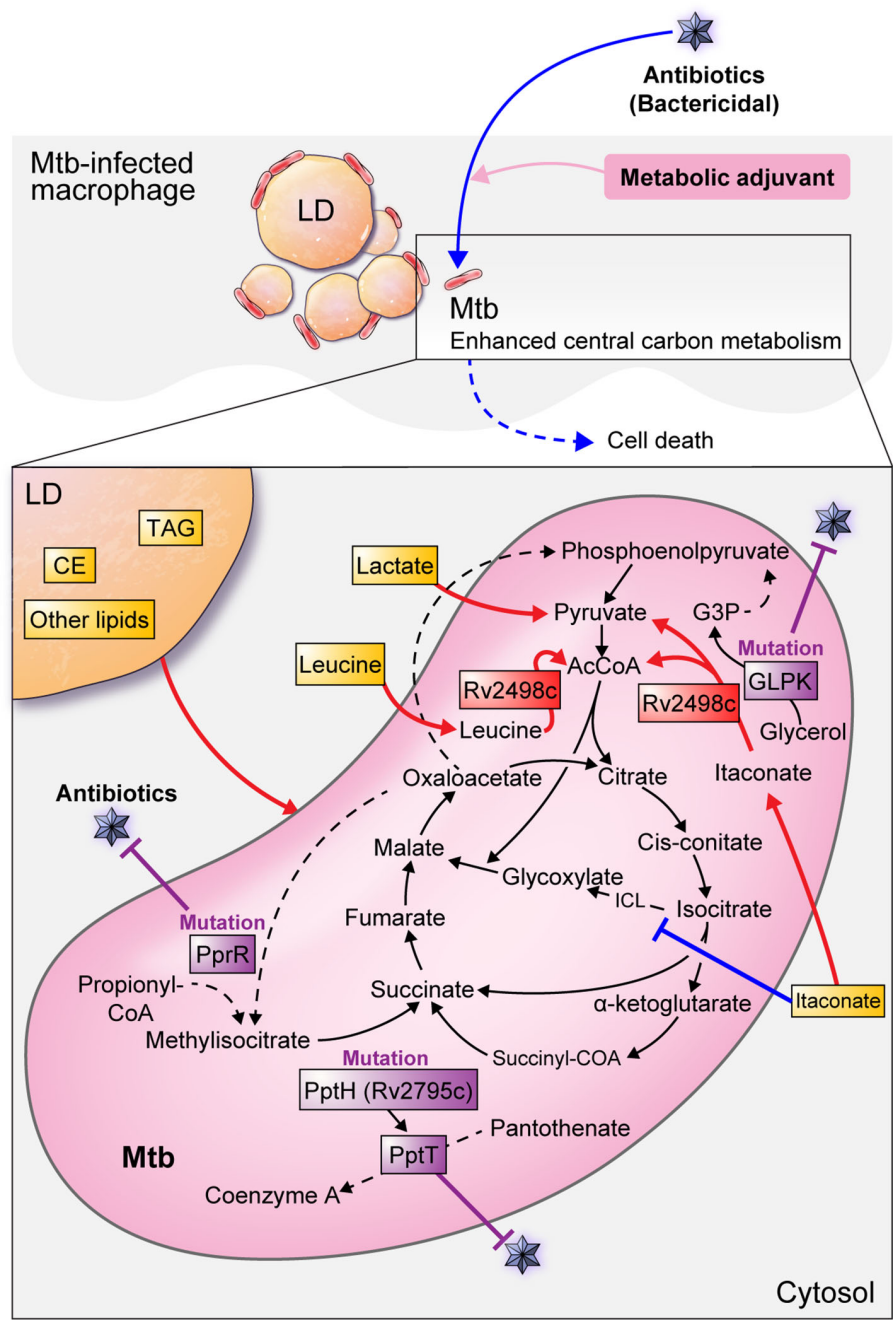
Mtb metabolism for survival and antibiotic defense in host cells. The Mtb survival strategy using host metabolism is shown as a red line. Mtb preferentially utilizes host pyruvate for intracellular proliferation. Similarly, lactate produced by glycolysis in the host is used for pathogens. Mtb Rv2498c decomposes itaconate to produce AcCoA and pyruvate, and leucine decomposes to produce AcCoA and acetoacetate, which are used as a nutrient source for Mtb. Lipid bodies in macrophages mainly consist of triacylglycerol (TAG) and cholesterol esters (CE) and can be a source of nutrients and components for Mtb. Inhibition of Mtb growth is shown as a blue line. Itaconate, a macrophage metabolite produced during the inflammatory response of Mtb-infected hosts, inhibits bacterial isocitrate lyse (ICL), a key enzyme in the glyoxylate cycle of mycobacteria. Antibiotic-resistant bacteria inhibit metabolism in antiseptic antibiotic treatment, but antibiotic susceptibility can be improved by using metabolic adjuvants to activate central carbon metabolism. Antibiotic resistance from altered Mtb metabolism and metabolism-related genes are shown as purple lines. Mutations in the *glpk* coding sequence reduce antibiotic efficacy and contribute to a drug-tolerant phenotype. *PrpR* mutations alter propionyl-CoA metabolism, resulting in attenuated antibiotic efficacy and induced multiple drug resistance. The Mtb killing effect is enhanced by an enzyme encoded with Ppt hydrolase (pptH) that hydrolyzes phosphopantetheinyl transferase (PptT) present in the CoA pathway. However, rv2795c loss-of-function mutations in Mtb confer resistance to antibiotics.

Itaconate, a macrophage metabolite produced during Mtb-infected host inflammatory responses, inhibits bacterial isocitrate lyase, a key enzyme in the glyoxylate cycle of mycobacteria (McFadden and Purohit, 1977; Michelucci et al., 2013; Moynihan and Murkin, 2014). However, a recent report found that Mtb participates in the itaconate dissimilation pathway and leucine catabolism to overcome host protective immune responses and to use host-derived antibacterial molecules as a nutrient source (Wang et al., 2019a). Mtb Rv2498c is a bifunctional enzyme that 1) dissimilates itaconate to produce AcCoA and pyruvate, and 2) catabolizes leucine to produce AcCoA and acetoacetate. In addition, Rv2498c deletion from the Mtb genome causes defects in the establishment of murine infection, indicating that dissimilation and catabolism by Rv2498c is critical for Mtb survival (Wang et al., 2019a). Additionally, a double mutant strain with Rv2498c (*CitE1*) and Rv3075c (*CitE2*) deletions, the β-subunits of citrate decomposition enzymes, showed a decrease in its growth in the lung and spleen of guinea pigs, indicating that the CitE enzyme may be a useful drug target (Arora et al., 2018). Collectively, researching how to inhibit the ability of Mtb to utilize host-derived molecules as a nutrient source could be a strategy to eliminate pathogens from the host (**Figure 3**) (**Table 2**).

### Mtb Persistence Mechanisms for Survival Strategies in the Host

In addition to antibiotic resistance and biofilm formation, the bacteria’s survival strategy form drug-resistant persistent cells to survive hostile environments or antibiotic stress (Fisher et al., 2017; Defraine et al., 2018). Mtb forms drug-resistant persistent cells to survive antibiotic stress (Torrey et al., 2016). Since virtually all antibiotics preferentially kill fast-replicating bacteria (Tomasz et al., 1970; Gomez and McKinney, 2004), quiescent metabolic activity and reduced growth contribute to “antibiotic-tolerance” (Baek et al., 2011; Harms et al., 2016). The reduction in metabolism of Mtb that contributes to the drug resistance phenotype is associated with the production of TAG (Baek et al., 2011). The *tgs1* of Mtb expressed during infection appears to have limited TCA cycle activity by using the acetyl-CoA carbon pool for TAG synthesis (Garton et al., 2008; Shi et al., 2010). When the *citA* gene is overexpressed, it competes effectively with acetyl-CoA, and when the TCA cycle is activated, TAG does not accumulate and the bacteria continue to grow (Baek et al., 2011). Additionally, whole genome sequencing and transcriptome analysis of a high persister Mtb mutant revealed that Mtb persister formation is related to genes in several pathways such as lipid biosynthesis, carbon metabolism, toxin-antitoxin systems, and transcriptional regulators (Torrey et al., 2016). In particular, Mtb in hypoxia conditions forms intracytoplasmic lipid inclusions (ILIs) using host-derived lipids to support persistence. ILIs serve to provide a carbon-based energy source that promotes dormancy in mycobacteria. Moreover, studies using two mycobacterial species with distinct lifestyles confirmed that a nitrogen-deficient and ILI-rich phenotype is associated with increased tolerance to several drugs used to treat mycobacteria infection (Santucci et al., 2019). Mtb can therefore play a role in controlling growth, metabolic rate, and antibiotic susceptibility by redirecting cellular carbon fluxes, as well as by providing a carbon storage function in preparation for long-term inactivity, by accumulating TAG (Daniel et al., 2004; Baek et al., 2011). Proteins other than those involved in bacterial metabolism are involved in the formation of persister cells of Mtb. The toxin-antitoxin (TA) system, widely distributed in Mtb, may be a promising therapeutic target, as it is an operon that modulates the adaptive response to the stress associated with the host environment and drug therapy (Solano-Gutierrez et al., 2019). Taken together, a new approach to eradicating persistent bacterial infection could be developed if the mechanism underlying persister cell formation and regrowth is better understood.

### Mtb Resistance Mechanisms for Survival Strategies in the Host

Unlike drug-tolerant persister cells, which are physiologically dormant, resistant strains may survive antibiotic treatment by having antibiotic resistance genes. In particular, mutations in genes related to Mtb metabolism can serve as a mechanism for responding to antibiotics by inducing Mtb persistence and drug resistance (Hicks et al., 2018; Bellerose et al., 2019). For example, *glpk* encodes the glycerol-3-kinase enzyme required for glycerol catabolism. Glycerol catabolism uses the lower glycolytic pathway to integrate into the anabolic pathway and spontaneously degrades methylglyoxal. *glpk* mutations are specific markers of multiple drug resistance in Mtb, and *glpk*-mutant strains contribute to Mtb persistence, drug-tolerance, and reduced antibiotic efficacy during treatment. (Bellerose et al., 2019). The synthesis of the Mtb cell wall and lipids important for toxicity relies on phosphopantetheinyl transferase (PptT) encoded by Rv2794c, so Mtb can be killed by inhibiting PptT. Mycobactericidal amidino-urea 8918 inhibits PptT, which is involved in the synthesis of cofactors such as CoA, by displacing the Ppt arm of CoA in the Ppt pocket. 8918 reduces the CFU of Mtb H37Rv *in vitro* and *in vivo* in mice, and exhibits antimycobacterial activity. In addition, the Mtb killing effect is enhanced by an enzyme encoded with Ppt hydrolase (PptH) that hydrolyzes PptT in the CoA pathway. However, loss-of-function in Rv2795c, which encodes PptH, confers resistance to 8918. As such, a mechanism that reduces PptT function in CoA metabolism using a PptH mutation in Mtb may be a mechanism that causes antibiotic resistance through a self-destructive reaction (Ballinger et al., 2019). In addition, mutations in the transcription factor *prpR* were found in drug resistant Mtb strains. *PrpR* mutations alter propionyl-CoA metabolism, resulting in attenuated antibiotic efficacy and multiple drug resistance. Furthermore, 1-5% of *prpR* mutations are present in drug-sensitive strains in various countries. Thus, even in the absence of drug resistance, *prpR* mutations could contribute to treatment failure (Hicks et al., 2018) (**Figure 3**) (**Table 2**).

## Using Metabolic Pathways to Develop Novel Therapeutic Strategies

### HDT Against Mycobacterial Infection by Controlling Metabolic Pathways

Since host cells and Mtb are extensively dependent on lipid and carbohydrate metabolic pathways, these metabolic pathways can be used as treatment targets (Shim et al., 2020). Formation of host foamy macrophages can persist during bacterial infection and contribute to the cavitation and release of infectious bacilli in patients with active disease (Pandey and Sassetti, 2008; Griffin et al., 2012). The ability to maintain chronic Mtb infection is associated with the ability of the host to use cholesterol. Statins inhibit MVA pathways in the host cholesterol biosynthesis pathway and enhance phagosomal maturation and autophagy (Parihar et al., 2014). Thus, statins can be targeted by host-directed drugs that induce protection against TB (**Figure 2**).

Macrophages infected with the virulent Mtb H37Rv strain preferentially synthesize lipoxin A4 (LXA_4_) using the precursor arachidonic acid, but do not synthesize PGE_2_. In contrast, macrophages infected with the Mtb H37Ra strain produce more PGE_2_, but less LXA_4_. Macrophages infected with the Mtb H37Rv strain that produce high LXA_4_ and low PGE_2_ undergo necrosis. In contrast, PGE_2_ production prevents necrosis and macrophage apoptosis. Infecting prostaglandin E synthase (PGES)-deficient macrophages *in vitro* with H37Rv shows a higher bacterial burden compared to wild-type macrophages. In addition, PGES^-/-^ mice showed significantly higher Mtb lung burden after infection with virulent Mtb. These results indicate that PGE_2_ plays a key role in inhibiting Mtb replication (Chen et al., 2008). Therefore, inhibiting the macrophage LXA_4_ synthesis pathway and inducing preferential PGE_2_ synthesis can inhibit Mtb replication in the host (**Figure 2**).

Arginine is beneficial as an adjuvant therapy in human immunodeficiency virus-negative patients with active TB. In the early stages of active TB treatment, arginine plays a role in enhancing human antimycobacterial defenses *via* increased iNOS-mediated NO production (Schon et al., 2003). Just as the adjuvant arginine improves the clinical outcome of TB patients, the development of HDT using metabolites may be a promising treatment for TB.

### Development of Metabolic Adjuvant Therapy to Enhance the Efficacy of Existing Antibiotics

Developing drug combinations based on bacterial metabolism is a predominant strategy to increase therapeutic efficacy, reduce drug toxicity, and prevent drug resistance. The TB drug BDQ is a species-selective Mtb ATP synthase inhibitor. The Mtb enzyme that responds most to ATP level was identified as glutamine synthetase (GS). The BDQ-mediated killing effect was increased by inhibiting Mtb GS. However, chemical supplementation with exogenous glutamine did not affect the antimycobacterial activity of BDQ. In other words, GS is not a direct antimycobacterial target, but a prime collateral vulnerability factor provided by BDQ. Thus, BDQ synergy combined with glutamine synthetase inhibitors presents a promising combinatorial approach to fight Mtb infection (Wang et al., 2019b).

Inhibiting bacterial metabolism using metabolite adjuvant can enhance the sensitivity of antibiotic resistant bacteria. To reduce aminoglycoside resistance in *E. coli* and *Staphylococcus aureus* populations, gentamicin was combined with metabolites found in upper glycolysis (glucose, mannitol, and fructose) and pyruvate. Indeed, gentamicin combined with the metabolite killed *E. coli* persister cells more effectively than gentamycin alone. These metabolites enhance aminoglycoside uptake by increasing proton-motive force by activating the electron transport chain (Allison et al., 2011). In another study, treating antibiotic-resistant *Psuedomonas aeruginosa* cells with a combination of various TCA cycle intermediates and tobramycin was reported to kill *P. aeruginosa* with increased efficacy (Meylan et al., 2017). Thus, developing metabolic adjuvants that enhance antibiotic effects by regulating bacterial metabolism using exogenous supplementation of certain metabolites may be a promising way to treat mycobacteria infections.

### Clinical Implications of Metabolism in TB Biomarkers

The development of biomarkers can help improve treatment and reduce medical costs. If a correlation between a disease and a biomarker can be established, the ability to diagnose and treat the disease will be greatly improved (Poste, 2011). For example, the American Society of Clinical Oncology estimates that testing colorectal cancer patients for the K-RAS tumor gene will save at least $600 million annually (Poste, 2011). Diagnosing diseases by metabolic profiling of patient biological samples and predicting the risk of disease progression can inform disease prevention and early treatment strategies (Feng et al., 2015; Lau et al., 2015; Cho et al., 2020). Certain metabolites in the plasma of patients with active TB have potential as biomarkers, and may reveal pathways involved in TB development and resolution (Weiner et al., 2018). In addition, metabolic markers in the altered host after completion of TB treatment may be associated with subsequent recurrent TB, and markers of host responses to treatment can facilitate the development of HDTs that can improve treatment efficacy (Qian et al., 2016).

Metabolites have long been clinically used as molecular markers (Yin and Xu, 2017). Since thousands of metabolites can be measured in an efficient and sensitive manner, metabolomics is used as a tool for the discovery of disease biomarkers (Dang et al., 2015). For example, metabolomics analysis can distinguish between Mtb and non-tuberculous mycobacterial infection by detecting six mycocerosates in patient sputum (Dang et al., 2015). In addition, methods for directly detecting Mtb-derived lipid components in sputum have been studied as diagnostic TB markers (Mourao et al., 2016), (Shui et al., 2012). However, people with human immunodeficiency virus or children have difficulty producing sputum samples (Kendall, 2017). In addition, individuals with asymptomatic TB, such as incipient or subclinical TB, do not cough and thus have difficulty in sputum-based testing (Drain et al., 2018).

Characteristic biomarkers or biometric detection methods are preferred for detecting active TB in non-sputum samples (Kik et al., 2014). Studies that distinguish active TB groups from controls show that metabolic pathways involving fatty acids, amino acids, and lipids in serum can identify active TB. In particular, a combination of lysophosphatidylcholine (18:0), behenic acid, threonyl-γ-glutamate, and free squalene diphosphate represents the biomarkers that are best suited to distinguish patients with TB activity from control subjects (Feng et al., 2015). In another study using patient plasma, 12R-hydroxy-5Z,8Z,10E,14Z-eicosatetraenoic acid, ceramide (d18:1/16:0), cholesterol sulfate, and 4α-formyl-4β-methyl-5α-cholesta-8-en-3β-ol levels were significantly higher in TB patients those in community-acquired pneumonia patients or controls (Lau et al., 2015). In addition, glutamate, sulfoxy methionine, and aspartate levels were higher, while glutamine, methionine, and asparagine levels were lower in the serum of TB patients than in individuals with latent TB infection or healthy controls. However, there were no significant differences in these metabolites according to the degree of disease or risk of relapse in patients with active TB (Cho et al., 2020). Since metabolism in the blood of a TB progressor significantly changes over time compared to that in the control group, the progression from infection to active TB can be predicted. Cortisol, kynurenine, glutamine, and histidine levels in the blood of TB progressors began to deviate from the control group approximately 6-12 months before active TB. In other words, it will be possible to prevent TB progression and transmission by analyzing the metabolic changes related to early symptoms observed up to 12 months before TB diagnosis. Thus, the temporal change in metabolite levels between TB progressors and the control group is important to identify patients with TB in progress (Weiner et al., 2018).

Additionally, bradykinin (BK) and desArg9-bradykinin (DABK) have been discovered as potential surrogate host response markers during early and late anti-TB treatment. Serum BK levels decrease during the early stages of anti-TB treatment and remain below baseline after the completion of treatment, while DABK levels tend to increase during the induction phase and decrease post-treatment. Elevated BK and DABK levels after the completion of treatment in TB patients may be associated with subsequent recurrent TB (Qian et al., 2016) (**Table 3**).

**Table 3:** Metabolic biomarkers of mycobacterial infection.

Biomarker	Study subjects	Purpose	Implications	Ref.
C26 and four mycocerosates	Sputum of 112 patients with TB	TB diagnosis	Positive correlation with TB patients	(Mourao et al., 2016)
Six mycocerosates	Sputum of 32 patients with TB from South Africa		Positive correlation with TB patients	(Dang et al., 2015)
MAs	Sputum of 70 patients with pulmonary TB		Positive correlation with TB patients	(Shui et al., 2012)
Trehalose-6-mycolate, phosphatidylinositol, resolvins	Plasma of 17 patients with TB disease and an asymptomatic household contact without TB disease		Largely upregulated in patients with TB disease	(Frediani et al., 2014)
LAM	Urine of 48 patients with TB		Positive correlation with TB patients	(Paris et al., 2017)
Tryptophan/kynurenine ratio with ADA	Serum of 156 patients with tuberculous pleurisy or malignant pleurisy	Distinguish TPE from MPE diseases	Lower tryptophan level and higher level of kynurenine in TPE	(Che et al., 2018)
Lipid metabolites including PG (16:0/18:1), LPI (18:0) and Ac1PIM1 (56:1)	Plasma of 17 adults with active pulmonary TB disease and 16 adults without active TB	TB diagnosis	Significantly increased in the active TB patients	(Collins et al., 2018)
3D, 7D, 11D-Phytanic acid, behenic acid, threoninyl-γ-glutamate	Serum of 146 patients with lung diseases that were due to non-TB conditions, and 120 patients with clinical signs of TB, 105 healthy	TB diagnosis	Decreased in active TB patients	(Feng et al., 2015)
Kynurenine, quinolinic acid, presqualene diphosphate			Significant upregulation in patients with active TB	
12(R)-HETE, ceramide (d18:1/16:0), cholesterol sulfate, and 4α-formyl-4β-methyl-5α-cholesta-8-en-3β-ol	Plasma of 46 patients with TB, 30 patients with community-acquired pneumonia, 30 controls without active infection	Diagnosis of TB	Significantly higher levels in TB patients than those in CAP patients and controls	(Lau et al., 2015)
Glutamate, sulfoxy methionine, and aspartate	Serum of 21 patients with active pulmonary TB, 20 subjects with LTBI, 28 healthy controls	Diagnosis of TB	Higher serum levels of metabolites in active TB patients than in LTBI subjects or healthy controls	(Cho et al., 2020)
Glutamine, methionine, and asparagine			Lower serum levels of metabolites in active TB patients than in LTBI subjects or healthy controls	
Cortisol, kynurenine	Blood of GC6-74 healthy, 4462 HIV-negative healthy household contacts of index TB progressors	Predicts TB progression	Higher abundances in the progressor group	(Weiner et al., 2018)
Glutamine, histidine			Lower abundances in the progressor group	
BK and DABK	Serum of 13 HIV-negative adults with microbiologically confirmed active TB	TB treatment response	Elevated BK and DABK levels after treatment completion	(Qian et al., 2016)

12(R)-HETE, 12R-hydroxy-5Z,8Z,10E,14Z-eicosatetraenoic acid; Ac1PIM1, acylphosphatidylinositol mannoside; ADA, adenosine deaminase; BK, bradykinin; DABK, DesArg^9^-bradykinin; CAP, community-acquired pneumonia; GC6-74, the grand challenges in global health GC6-74 project; HIV, human immunodeficiency virus; LAM, lipoarabinomannan; LPI, lysophosphatidylinositol; LTBI, latent tuberculosis infection; MAs, mycolic acids; MPE, malignancy pleural effusion; PG, phosphatidylglycerol; TB, tuberculosis; TPE, tuberculous pleural effusion.

## Conclusions and Prospective

Recent advances in the field of host–pathogen interactions have emphasized the importance of mutual metabolic reprogramming, not only to understand the mechanisms of drug tolerance but also to develop effective therapeutic strategies. Metabolic modulation of immune cells by infection with mycobacterial pathogens may be a crucial decision-making step, eventually leading to differential disease outcomes from pathogen clearance to severe disease progression by regulating host-favoring or pathogen-favoring conditions. Thus, mycobacterial pathogenicity may be directly associated with the ability to shift host metabolism towards pathogen-favoring conditions by reprogramming not only pathogenic factors, but also glycolysis, the TCA cycle, fatty acid metabolism, and nitrogen metabolism in host immune cells.

This review focuses primarily on the metabolism of immune cells and Mtb and provides a general overview of Mtb pathogenesis and progression through studies of its role in metabolic reprogramming. Macrophages infected with Mtb may activate immune responses and metabolic programming to defend against pathogens, but these changes also lead to immune-resistant pathogens. Pathogenic bacteria use various adaptation strategies, such as using host-derived metabolites as nutrients to survive inside host cells or regulating bacterial and host metabolism to resist antibiotics. Although it is becoming increasingly apparent that proper metabolic activation of macrophages is necessary to control Mtb infection, research into strategies to improve treatment outcomes using bacterial and host metabolic regulation is still ongoing. For innovative development of therapeutic agents to treat infectious Mtb diseases, it is necessary to understand the metabolism crucial for the protection of hosts infected with mycobacteria versus the metabolism that favors pathogen survival. Developing HDT using immunomodulatory control to protect an infected host and improve the ability to diagnose and treat disease is a major attempt to improve clinical outcomes in the treatment of lung infections. Mtb has a strategy of forming persister cells that can survive exposure to antibiotics by maintaining a low metabolic state. Further ways to improve the metabolic activity and drug sensitivity by redirecting the bacterial TAG synthesis pathway to the TCA cycle to avoid this quiescent metabolic activity is a major goal of TB research. In addition, attempts to increase antibiotic efficacy using metabolic adjuvant therapy to control host and resistant bacterial metabolism when an antibiotic is given to an infected host may help design new treatment strategies.

## Author Contributions

J-HP, DS, KK, WL, and SS wrote the manuscript. SS conceived the study, supervised the team and critically revised the manuscript. All authors contributed to the article and approved the submitted version.

## Funding

This work was supported by the National Research Foundation of Korea (NRF) grant (NRF-2019R1A2C2003204) and the Bio & Medical Technology Development Program of NRF (NRF-2020M3A9H5104234) funded by the Korea government (MSIT), Republic of Korea. The funders had no role in study design, data collection and analysis, decision to publish, or preparation of the manuscript.

## Conflict of Interest

The authors declare that the research was conducted in the absence of any commercial or financial relationships that could be construed as a potential conflict of interest.
